# Delayed right ventricular perforation 3 years after permanent pacemaker implantation: a case report

**DOI:** 10.1097/RC9.0000000000000138

**Published:** 2026-02-03

**Authors:** Sinh Hien Nguyen, Thanh Hung Ngo, Pham Thi Hue

**Affiliations:** a Department of Adult Cardiac Surgery, Hanoi Heart Hospital, Hanoi, Vietnam; b Faculty of Medicine Ramathibodi Hospital, Mahidol University, Bangkok, Thailand

**Keywords:** cardiac tamponade, late lead perforation, lead extraction, pacemaker complication, right ventricular perforation

## Abstract

**Introduction:**

Right ventricular (RV) lead perforation is an uncommon but serious complication of pacemaker implantation. Most cases occur acutely or subacutely, while very late perforations – beyond 1 year – are exceedingly rare and often unexpected.

**Presentation of case:**

We report the case of a 53-year-old woman who presented with syncope and cardiac arrest 3 years after permanent pacemaker implantation. Imaging revealed cardiac tamponade due to RV lead perforation. The patient underwent emergency median sternotomy, lead extraction, and RV repair without the use of cardiopulmonary bypass. Postoperatively, spontaneous sinus rhythm was preserved, and pacemaker reimplantation was not required.

**Discussion:**

Very late lead perforation is rarely reported, especially in young patients without risk factors. This case highlights the unpredictable nature of the complication, the value of contrast-enhanced CT in diagnosis, and key surgical considerations, including when bypass may be avoided and when reimplantation is unnecessary.

**Conclusion:**

This case emphasizes the importance of long-term surveillance in pacemaker recipients and individualized management strategies for delayed RV lead perforation.

## Introduction

Right ventricular (RV) lead perforation is a rare but potentially life-threatening complication of pacemaker implantation, with a reported incidence of 0.1–0.8% for pacemakers and up to 5.2% for implantable cardioverter-defibrillators (ICDs)^[^1,2^]^. Most cases occur within 30 days post-implantation, while delayed perforations are defined as those occurring beyond 1 month are exceedingly uncommon and often present with nonspecific symptoms such as chest pain, dyspnea, or extracardiac stimulation.^[^3–5^]^

Risk factors include older age, female sex, steroid use, and active fixation leads, although cases have also been reported in younger patients without these factors^[^3,4^]^. Diagnosis can be challenging; while transthoracic echocardiography and chest radiographs may be inconclusive, computed tomography (CT) offers superior diagnostic accuracy^[^2,5^]^.

We report a rare case of delayed RV perforation occurring 3 years after pacemaker implantation in a relatively young woman without classical risk factors. To the best of our knowledge, no cases of delayed right ventricular perforation have previously been documented in our country. The case highlights the need for long-term vigilance and prompt recognition of this rare but serious complication. This case report has been prepared in accordance with the revised Surgical CAse REport (SCARE) 2025 guidelines^[^6^]^.

**HIGHLIGHTS**

- Right ventricular perforation can occur very late after pacemaker implantation.
- Our case involved a 53-year-old female, much younger than most reported cases.
- The lead perforated through the right ventricular apex causing massive pericardial effusion.
- Surgical intervention was life-saving; no pacemaker reimplantation was necessary.

## Case presentation

A 53-year-old woman had undergone permanent pacemaker implantation 3 years earlier for second-degree atrioventricular block. After implantation, she experienced recurrent episodes of chest discomfort. One month prior to admission, she began to feel increasingly fatigued, developed lower limb edema, and had decreased urine output. She was evaluated at another medical facility and diagnosed with pericardial effusion, suspected to be Dressler’s syndrome. Pericardiocentesis was performed, draining 500 ml of non-clotting blood. Tests for tuberculosis and malignancy were negative. She was treated with non-steroidal anti-inflammatory drugs (NSAIDs) for one week and discharged.

Two days after discharge, she experienced a syncopal episode followed by cardiac arrest, from which she was successfully resuscitated. Upon admission to our hospital, she was conscious but fatigued, with dyspnea corresponding to NYHA class II–III. She also presented with peripheral edema, hepatomegaly, and anemia.

**Figure 1.**
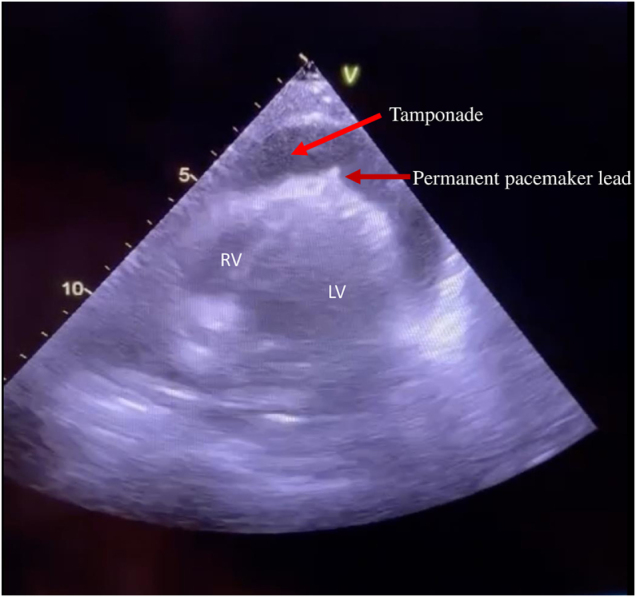
Transthoracic echocardiography showing massive pericardial effusion compressing the right ventricle. The pacemaker lead is seen protruding outside the pericardial sac.

Transthoracic echocardiography revealed a large amount of free pericardial effusion causing cardiac tamponade. The pacemaker lead appeared to be protruding through the right ventricular apex (Fig. 1).

**Figure 2.**
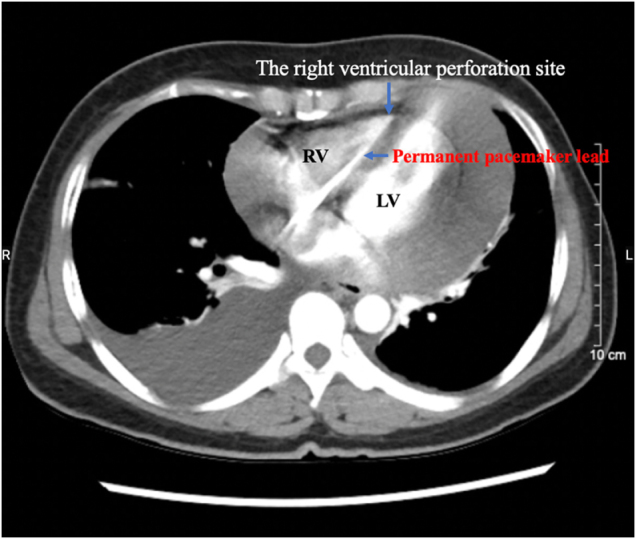
Contrast-enhanced computed tomography confirming the presence of pericardial effusion causing cardiac tamponade. The pacemaker lead is visualized perforating through the right ventricular wall.

Contrast-enhanced computed tomography confirmed the presence of a large pericardial effusion with tamponade and demonstrated the pacemaker lead traversing the right ventricular wall (Fig. 2).

**Figure 3.**
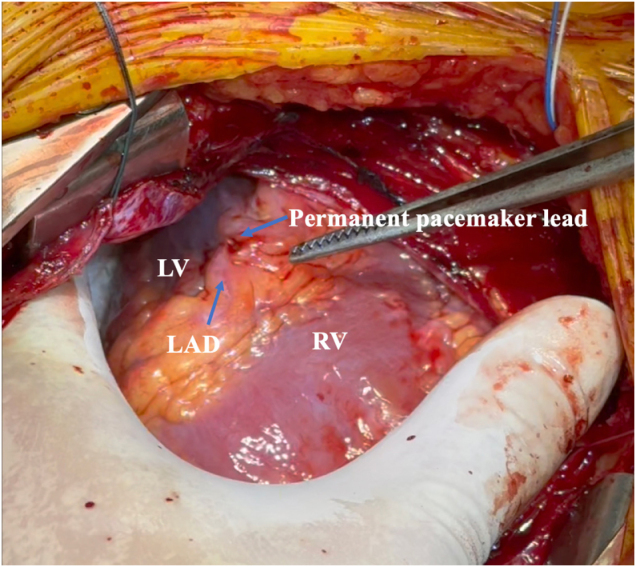
Intraoperative findings showing the pacemaker lead penetrating through the right ventricular apex, located adjacent to the left anterior descending artery, extending approximately 0.5 cm beyond the epicardial surface.

**Figure 4.**
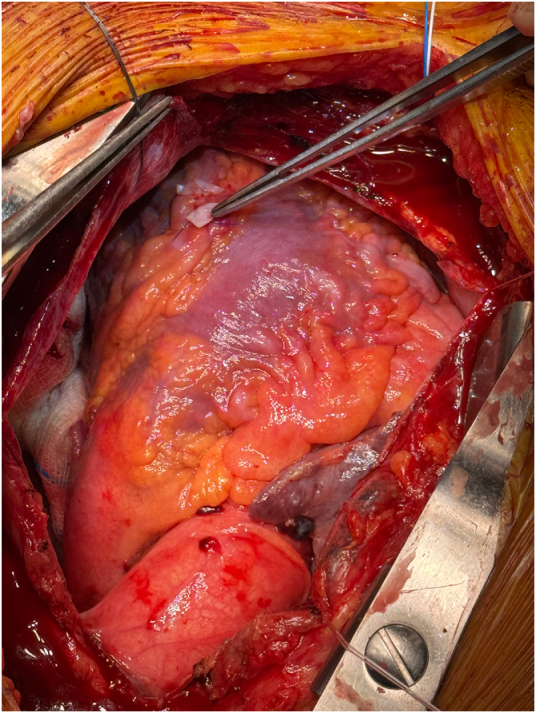
The perforation site on the right ventricle was closed with sutures reinforced by a pericardial patch on both sides.

The patient underwent emergency surgery. Upon opening the pericardium, 600 ml of non-clotting blood was evacuated. The pacing lead was found to have perforated through the apex of the right ventricle, lying approximately 0.5 cm outside the epicardium, adjacent to the left anterior descending artery (Fig. 3). The pacemaker generator and lead were removed. The myocardial perforation site was closed with sutures reinforced by a pericardial patch (Fig. 4; https://drive.google.com/file/d/1huioygh6jfdcdkqjxahbkberx8v98opl/view?usp=sharing, video).

Postoperatively, the patient was extubated after 2 hours, and the postoperative ICU stay was 1 day, with no additional complications observed. A 24-hour Holter monitoring confirmed sinus rhythm, with no atrioventricular block or pauses longer than 2 seconds. Therefore, pacemaker reimplantation was deemed unnecessary. The patient was discharged 1 week later in stable condition.

## Discussion

Right ventricular (RV) lead perforation is a rare but potentially fatal complication of permanent pacemaker or implantable cardioverter-defibrillator (ICD) implantation. Its reported incidence ranges from 0.1 to 0.8% for pacemakers and may reach up to 5.2% for ICDs, depending on device type, lead characteristics, and patient factors^[^1,2^]^. Lead perforation is temporally classified into acute (<24 hours), subacute (1–30 days), and delayed or chronic (>30 days). While most cases are detected early, very late presentations – occurring years after implantation – are extremely uncommon and clinically unpredictable^[^3,4,7^]^.

The pathophysiology of delayed perforation is thought to involve chronic micro-motion of the lead tip against the endocardium, leading to progressive myocardial erosion. Over time, this mechanical irritation may be exacerbated by myocardial thinning, local inflammation, or fibrosis. Although active fixation leads are traditionally considered higher risk, passive fixation leads have also been implicated, particularly when positioned at the RV apex^[^3,8^]^. To prevent lead migration and free right ventricular wall perforation, the lead should be anchored securely into the interventricular septum and positioned away from the apex.

Our patient’s case is striking for its timing and severity: RV perforation occurred 3 years after pacemaker implantation, presenting as sudden syncope and cardiac arrest due to tamponade. This stands in stark contrast to most reports of delayed perforation, which tend to occur within the first year and present more insidiously. In a review of 54 cases by Issa *et al*, the average delay was approximately 60 days, and nearly half of patients were asymptomatic[4].

Although very late lead perforation is rare, certain precipitating factors may accelerate the erosion process. Documented risks include chronic corticosteroid use, long-term anticoagulation, low body mass index, renal insufficiency, recent chest trauma, and device infection^[^3,8^]^. Some case reports suggest that minor intrathoracic pressure changes – due to coughing or physical strain – may also precipitate lead migration in vulnerable tissue[9]. However, our patient had no identifiable risk factors, no recent trauma or infection, and no history of steroid or anticoagulant use.

In some cases, progressive lead impedance decline may signal early migration due to insulation failure or tip micro-displacement[10]. However, in our patient, device interrogations remained stable until the acute event. Interestingly, she reported intermittent chest discomfort for several months, which – though nonspecific – may have represented a subtle prodrome that was initially overlooked. Therefore, in patients with a history of pacemaker implantation who present with chest pain in the absence of myocardial infarction, lead migration should be carefully evaluated.

Compared to prior reports, our case is particularly striking. A comparison of previously published cases of delayed right ventricular lead perforation, including patient characteristics, timing, and management strategies, is summarized in Table 1. Yamamoto *et al* described a 2-year delayed perforation in a 77-year-old woman without tamponade, managed conservatively[5]. Madanat *et al* reported a 9-month delayed case presenting with phrenic nerve stimulation and treated percutaneously[11]. Iwai *et al* documented an 8-year delayed perforation of a passive lead, but the patient was hemodynamically stable[9]. In contrast, our patient was younger (53 years), had no conventional risk factors, no parameter drift, and presented with acute tamponade and cardiac arrest – highlighting the unpredictability and severity of this complication.

**Table 1:** Comparative summary of published cases of delayed right ventricular lead perforation.

Author/Year	Patient age	Interval to RV perforation	Cardiac tamponade	Management
Our patient	53-year-old female	3 years	Yes	Emergency sternotomy; pericardial decompression; direct lead extraction; RV repair with pledgeted sutures and pericardial patch; no CPB; temporary epicardial pacing; no reimplantation
Yamamoto *et al*, 2022	67-year-old female	2 years	No	Elective surgery; RV wall repair; CPB used; new RV lead implanted
Iwai *et al*, 2023	89-year-old male	8 years	No	Hybrid: minimally invasive thoracotomy + transvenous extraction; RV repair without patch; new active-fixation lead; no CPB

Diagnosis of lead perforation remains challenging. While transthoracic echocardiography (TTE) is often the first imaging modality, it may fail to identify the exact trajectory of the lead. Chest radiography also has low sensitivity. Computed tomography (CT), by contrast, provides superior spatial resolution and diagnostic accuracy. In a study by Rajkumar *et al*, CT had 92.9% sensitivity and 85.7% specificity in detecting lead perforation[2]. In our case, contrast-enhanced CT was essential for confirming the perforation and guiding surgical planning. However, in cases of acute tamponade, MSCT should only be performed when the patient’s hemodynamics are stabilized or when differentiation from acute aortic pathology is required.

Surgical management was carried out through a median sternotomy, providing optimal exposure for rapid pericardial decompression and precise control of the perforation site. Approximately 600 ml of non-clotting pericardial blood was evacuated, confirming tamponade physiology. The lead was visualized protruding through the anterior right ventricular apex, extending approximately 0.5 cm beyond the epicardium, and notably positioned near the left anterior descending (LAD) artery. This proximity necessitated meticulous dissection to avoid iatrogenic injury to epicardial coronary branches.

After decompressing the pericardium and stabilizing the patient, the pacing lead was gently extracted under direct vision, with counter-traction applied from the generator pocket to avoid myocardial avulsion or further tissue damage. The entire pacemaker system was removed en bloc. The RV defect was closed using pledgeted horizontal mattress sutures with non-absorbable polypropylene, and reinforced with an autologous pericardial patch to distribute tension and support hemostasis. Use of a biologic adhesive sealant was considered but ultimately not required due to adequate suture line control. After removal of the pacemaker system, the patient remained in sinus rhythm without evidence of conduction abnormalities; therefore, we elected to place a temporary epicardial pacing lead rather than proceeding with immediate permanent pacemaker reimplantation.

In cases of right ventricular perforation caused by pacemaker leads, the defect is typically small and surrounded by relatively firm myocardium; therefore, cardiopulmonary bypass is often not required for repair. In contrast to several previous reports where CPB was preemptively established – particularly when the lead was embedded deeply, the myocardium was friable, or tamponade was severe – we achieved hemodynamic stabilization and successful repair without bypass^[^5,11^]^. This supports the notion that in well-selected cases with localized injury and experienced surgical teams, CPB may be avoided.

Postoperatively, the patient was monitored in the hospital for 7 days after surgery, and 24-hour Holter recording were performed to evaluate native conduction. As sinus rhythm remained stable without pauses or conduction disturbances, reimplantation was deemed unnecessary. While prior cases in the literature proceeded with permanent pacemaker reimplantation regardless of rhythm status, our decision to withhold reimplantation reflects a more individualized, physiology-driven approach^[^5,9^]^. It also underscores the importance of reassessing pacing dependency, especially when the initial indication was non-mandatory or potentially transient. At the 6-month postoperative follow-up, the patient remained in stable sinus rhythm and had no clinical symptoms.

These procedural details – particularly the avoidance of CPB and the non-reimplantation strategy – distinguish our case from many others and may inform future decision-making in similar clinical scenarios.

## Conclusion

Very late right ventricular lead perforation is a rare but potentially life-threatening complication that may present abruptly, even in the absence of conventional risk factors or warning signs. This case underscores the importance of long-term vigilance in pacemaker recipients and highlights the diagnostic value of contrast-enhanced CT in acute presentations. Surgical intervention without cardiopulmonary bypass can be effective in selected cases, and the need for device reimplantation should be reassessed based on individualized conduction status. Early recognition and tailored management remain key to improving outcomes in this rare but serious complication.

## Data Availability

The data supporting the findings of this study are available from the corresponding author upon reasonable request.

## References

[1] Hsu JC Varosy PD Bao H. Cardiac perforation from implantable cardioverter-defibrillator lead placement. Circ Cardiovasc Qual Outcomes 2013;6:582–90. 24002030 10.1161/CIRCOUTCOMES.113.000299

[2] Rajkumar CA Claridge S Jackson T. Diagnosis and management of iatrogenic cardiac perforation caused by pacemaker and defibrillator leads. Europace 2017;19:1031–37. 27353321 10.1093/europace/euw074

[3] Refaat MM Hashash JG Shalaby AA. Late perforation by cardiac implantable electronic device leads: clinical presentation, diagnostic clues, and management. Clin Cardiol 2010;33:466–75. 20734443 10.1002/clc.20803 PMC6653135

[4] Issa ZF Issa TZ. Feasibility and safety of percutaneous lead revision for subacute and delayed cardiac device lead perforation. JACC Clin Electrophysiol 2021;7:26–35. 33478709 10.1016/j.jacep.2020.07.024

[5] Yamamoto A Takahashi S. Delayed right ventricular lead perforation by a pacemaker lead 2 years post-implantation. Clin Case Rep 2022;10:e05760. 35449773 10.1002/ccr3.5760 PMC9014697

[6] Kerwan A Al-Jabir A Mathew G. Revised Surgical CAse REport (SCARE) guideline: an update for the age of artificial intelligence. Prem J Sci 2025;10:100079.

[7] Ellenbogen KA Wood MA Shepard RK. Delayed complications following pacemaker implantation. Pacing Clin Electrophysiol 2002;25:1155–58. 12358163 10.1046/j.1460-9592.2002.01155.x

[8] Mahapatra S Bybee KA Bunch TJ. Incidence and predictors of cardiac perforation after permanent pacemaker placement. Heart Rhythm 2005;2:907–11. 16171740 10.1016/j.hrthm.2005.06.011

[9] Iwai H Takami M Fukuzawa K. Very late perforation of a passive fixation lead 8 years after pacemaker implantation. HeartRhythm Case Rep 2023;9:351–54. 37361986 10.1016/j.hrcr.2023.02.014 PMC10285091

[10] Hauser RG Kallinen LM Almquist AK. Early failure of a small-diameter high-voltage implantable cardioverter-defibrillator lead. Heart Rhythm 2007;4:892–96. 17599673 10.1016/j.hrthm.2007.03.041

[11] Madanat L Shah K Bloomingdale R. Diaphragmatic pacing as an initial presentation of delayed ventricular lead perforation. J Innov Card Rhythm Manag 2022;13:5004–08. 35655806 10.19102/icrm.2022.130504 PMC9154013

